# The Effect of Preincubation Time and Myo-inositol Supplementation on the Quality of Mouse MII Oocytes

**DOI:** 10.18502/jri.v21i4.4330

**Published:** 2020

**Authors:** Fatemeh Mohammadi, Mahnaz Ashrafi, Zahra Zandieh, Mohammad Najafi, Behrooz Niknafs, Fatemeh Sadat Amjadi, Maryam Haghighi

**Affiliations:** 1- Student Research Committee, School of Medicine, Iran University of Medical Sciences (IUMS), Tehran, Iran; 2- Anatomy Department, School of Medicine, Iran University of Medical Sciences (IUMS), Tehran, Iran; 3- Shahid Akbar Abadi Clinical Research Development Unit (ShACRDU), Iran University of Medical Sciences (IUMS), Tehran, Iran; 4- Cellular and Molecular Research Center, Iran University of Medical Sciences (IUMS), Tehran, Iran; 5- Biochemistry Department, School of Medicine, Iran University of Medical Sciences (IUMS), Tehran, Iran; 6- Anatomy Department, School of Medicine, Tabriz University of Medical Sciences, Tabriz, Iran

**Keywords:** Developmental rate, Fertilization potential, Mitochondrial alteration, Myo-inositol supplement, Oocyte preincubation time, Oocyte quality, Oxidative stress

## Abstract

**Background:**

It is demonstrated that optimal preincubation time improves oocyte quality, fertilization potential and developmental rate. This study aimed to evaluate the effect of preincubation time in the simple and myo-inositol supplemented medium on the oocyte quality regarding oxidative stress and mitochondrial alteration.

**Methods:**

Cumulus oocyte complexes (COCs) retrieved from superovulated NMRI mice were divided in groups of 0, 4 and 8 *hr* preincubation time in the simple and 20 *mmol/L* myo-inositol supplemented media. Intracellular reactive oxygen species (H_2_O_2_), glutathione (GSH), mitochondrial membrane potential (MMP), ATP content, and mitochondrial amount were measured and analyzed in experimental groups. One-way ANOVA and Kruskal-Wallis were respectively used for parametric and nonparametric variables. Statistical significance was defined as p<0.05.

**Results:**

In comparison to control group, variables including ROS, GSH, mitochondrial amount, fertilization and developmental rates were significantly changed after 4 *hr* of preincubation in the simple medium, while MMP decreased following 8 *hr* of preincubation in the simple medium (p˂0.001). Preincubation of oocytes up to 8 *hr* in the simple medium could not decrease ATP content. For both 4 and 8 *hr* preincubation times, myo-inositole could decrease H_2_O_2_ and increase GSH and MMP levels and consequently could improve fertilization rate compared to oocytes preincubated in the simple culture.

**Conclusion:**

It seems that 4 *hr* or more preincubation time can decrease the oocyte quality and lead to reduced oocyte fertilization and developmental potential. Howevere, myo-inositol may prevent oocyte quality reduction and improve fertilization potential in comparision to the equivalent simple groups.

## Introduction

Preincubation is the temporary cultivation of oocytes before assisted reproductive technology (ART) procedures, in which the retrieved oocytes are kept in the incubator at 37*°C* with 5 to 6% of CO_2_ (1). There are controversial results regarding the effects of different preincubation times on the clinical outcomes such as fertilization, cleavage rate, blastocyst formation, embryo quality, implantation and pregnancy rate (2–8). In addition, preincubation time recommended by some articles is challenged by the other studies leading to the failure to define a standard preincubation time (9–11).

Since oocytes require more time for cytoplasmic maturation compared to nuclear maturation, it seems that oocyte preincubation results in a synergistic effect on nuclear and cytoplasmic maturation (12, 13). On the other hand, extending the culture of oocytes before *in vitro* fertilization/intracytoplasmic sperm injection (IVF/ICSI) could have a negative effect due to the postovulatory aging of the oocytes (14). Postovulatory oocyte aging as a time -dependent reduction in oocyte quality is related to decreased fertilization rate, developmental potential, embryo quality and pregnancy rate and therefore it limits the success of assisted reproductive techniques (14–16).

Although the main mechanism of this decline in oocyte quality is not fully understood, oxidative stress and mitochondrial changes have been suggested as effective factors (17). The mitochondria are the main organelles of the oocyte, which are involved in adenosine triphosphate (ATP) production, calcium homeostasis and signaling, reactive oxygen species (ROS) generation and cell apoptosis (18). The mitochondrial function is considered as an important marker for the quality and developmental potential of the oocyte (19, 20). Oocyte aging is associated with an increase in intracellular ROS, lack of antioxidants, variation in the mitochondrial number and distribution, impaired mitochondrial membrane potential (MMP), and a decrease in ATP level (17, 21–24).

Inositol is one of the important components of the structural fatty acids. Structural fatty acids like phosphatidylinositol are the main compositions of the cellular membranes, specially mitochondrial membrane and play a crucial role in the membrane stability and function of mitochondria (25). Myo-inositol (MYO), as the most important form of inositol in nature, participates in the cell morphogenesis and cytogenesis, cell membrane formation, lipid synthesis and cell growth. MYO is considered as a precursor of second messengers in the cellular signal transduction system and consequently involves in the regulation of intracellular calcium concentration (26, 27). Therefore, it plays an important role in the cardiac regulation, insulin sensitization, metabolic alterations and particularly reproduction (28–31). On the other hand, it has been demonstrated that MYO has an antioxidant effect and reduces the oxidative stress (32, 33). MYO in body fluids especially in the follicular fluid is responsible for the generation of important intracellular signals. It is also essential for follicular maturation and acts as a marker of good quality in oocytes (34–36). Nowadays, additional nutrients including myo-inositol are recommended for therapeutic support of oocyte health and fertility in women of advanced reproductive age (37).

To date, available clinical studies have not found a clear boundary between optimal and undesirable oocyte preincubation time. On the other hand, in spite of the positive effect of MYO on the mitochondrial function and its antioxidant properties, there is no data on the effects of MYO supplementation on the oocyte quality preservation during the oocyte culture. Therefore, this research aimed to evaluate the effect of preincubation time in the simple and MYO supplemented medium on the intracellular ROS and glutathione levels, amount of mitochondria, mitochondrial membrane potential, ATP content, fertilization and developmental rates in the mouse MII oocytes, as a step in determination of appropriate preincubation condition in ART clinics.

## Methods

### Chemical and media:

The handling of the retrieved oocytes was performed outside the CO_2_ incubator in M2 medium supplemented with 0.5% bovine serum albumin (BSA). The culture medium used for oocyte preincubation was Eagle's Minimum Essential Medium (MEM). Most of the chemical reagents and also medium used in this study were supplied from Invitrogen Co. (Carlsbans, California, USA) unless otherwise stated.

### Ethical approval:

All procedures dealing with mice were done according to the criteria presented in the National Institutes of Health guidelines for Care and Use of Laboratory. The experimental protocol was approved by the Animal Ethics Committee of University with the number IR. IUMS.FMD.REC 1396.9321113002.

### Animals:

Six to eight week -old adult female and 8 to 10 week–old male NMRI mice were used in this study. Mice were housed under a 12/12 light and dark regimen, at 22 to 24*°C* and 40 to 50% of humidity, with food and water available ad libitum.

### Oocyte retrieval and preincubation:

In order to collect MII oocytes, adult female mice were superovulated by injecting 7.5 *IU* of Folligon pregnant mare serum gonadotrophin intraperitoneally (PM-SG, MSD, Intervet, Australia) followed by 10 *IU* of human chorionic gonadotropin (hCG, Darou Pakhsh, Tehran, Iran) 50 *hr* later. Almost 16 to 17 *hr* after hCG injection, cumulus-oocyte complexes (COCs) were collected from oviductal ampulla and divided randomly into five experimental groups: (1) no preincubation/control, (2) 4 *hr* preincubation (Half of the work shift duration in the clinic) in simple medium, (3) 4 *hr* preincubation in 20 *mmol/L* of myo-inositol supplemented medium, according to Chiu et al.’s study (38), (4) 8 *hr* preincubation (Full duration of the work shift) in simple medium, (5) 8 *hr* preincubation in 20 *mmol/L* of myo-inositol supplemented medium. After completion of the preincubation period, the cumulus cells were removed by mechanical pipetting following brief incubation in 0.2% of hyaluronidase (Sigma-Aldrich, Louise, Missouri, USA). Denuded oocytes were observed carefully under a stereo microscope. Oocytes with a clear polar body were considered to be used for further analysis.

### Analysis of intracellular ROS (H_2_0_2_) levels:

The intracellular H_2_O_2_ level in oocytes was analyzed using 2′, 7′-Dichlorodihydrofluorescein diacetate (H2DCFDA) dye. At each iteration, 20 MII oocytes from each group were incubated for 30 *min* at 37°*C* in phosphate-buffered saline containing 10 *μM* of H2DCFDA (39). Oocytes were washed three times using BSA-supplemented PBS and were placed in cell culture dishes. Then, fluorescence intensity was measured using inverted fluorescence microscopy (Olympus, Tokyo, Japan) with 450–490 *nm* filters. Considering the probability of missing the samples during the staining process, analysis was repeated at least 3 times and finally the fluorescence pixel intensity of 40 oocytes was analyzed using ImageJ software (Version 1.50; National Institutes of Health, Bethesda, MD, USA).

### Detection of intracellular GSH (glutathione) levels:

Cell Tracker Blue CMF2HC (4-chloromethyl-6,8-difluoro-7-hydroxycoumarin) fluorochrome was used to analyze the intracellular GSH levels. To do this, at each experiment, 20 MII oocytes from each group were incubated for 30 *min* at 37°*C* in phosphate-buffered saline containing 10 *μM* of Cell Tracker Blue (40, 41). Oocytes were washed three times by BSA-supplemented PBS and were placed in cell culture dishes. Analysis was repeated 3 times and fluorescence intensity of 40 oocytes was measured using inverted fluorescence microscopy with 370 *nm* filters. Image J software was used to analyze the fluorescence intensity of the oocytes.

### Analysis of amount of mitochondria:

20 MII oocytes from each experimental group were stained with 0.2 *mM* of Mito Tracker Green fluorochrome for 5 *min* at 37*°C* (42). After washing them three times by gamete buffer (Cook, Bloomington, Indiana‎, USA), the oocytes were placed in glass bottom cell culture dishes and were observed under an inverted fluorescence microscope with 490 *nm* filters. After 3 replications, the fluorescence intensity of the 40 oocytes was quantified and analyzed by Image J software.

### Determination of MMP:

20 MII oocytes from each group were cultured in MEM medium containing 2 *µM* of JC-1 dye (Mitochondrial Membrane Potential Probe) for 30 *min* at 37°*C* (43). After washing the oocytes by MEM medium, they were placed in the cell culture dishes. Filters were set at 488 *nm* and 525 *nm* for green and red fluorescence, respectively. The fluorescence intensity in each oocyte was measured under a fluorescence microscope with the same scan setting for each sample. The analysis was repeated at least 3 times and mitochondrial membrane potential of 40 oocytes was assessed based on the ratio of the red/green fluorescence intensity following the analysis of the fluorescence pixel intensity using Image J software.

### ATP content assay:

In order to assess the ATP content, at each iteration, 15 MII oocytes from each group were loaded in the microtubes using 20 *μl* of ultrapure water and were stored at −80*°C* until assayed. The measurement of ATP in the oocytes was performed by a colorimetric-based commercial mouse adenosine triphosphate ELISA kit (ZellBio GmbH, Germany). Oocytes were mixed by 100 *µl* of schizolysis solution and were vortexed for 1 *min* on ice for obtaining the lysis, then the mixture was centrifuged at 10000×*g* for 10 *min* at 4°*C* and supernatant was collected to be used. Then, reagents were added sequentially to the samples according to the manufacturer’s instructions. A 6-point standard curve (0 to 5 *ng/ml*) was included in each assay. Standard curves were generated and the ATP content was measured using formula derived from the linear regression of the standard curve.

### In vitro fertilization:

During IVF procedure, following centrifugation of sperm collected from the cauda epididymis of male mice at 300×*g* for 5 *min* in 1000 *μl* of HTF medium (Global Life, McKinney, USA), the pellet was incubated in 400 *μl* of HTF medium for 60 *min* at 37°*C*. Afterwards, 5 COCs were transferred to 50 *μl* droplets of COOK fertilization medium (Bloomington, Indiana‎, USA) and swim-up sperm was added to the droplets containing COCs. After 4 to 5 *hr* of co-incubation of COCs and sperm, oocytes were recovered and washed three times in HTF medium to remove the remaining spermatozoa and assessed in terms of pronucleus formation. The developmental potential of fertilized oocyte up to 8-cell stage, was examined following 3 days of culturing the fertilized oocytes in the 20 *μl* droplets of COOK cleavage medium (Bloomington, Indiana‎, USA). The experiment was repeated to evaluate fertilization and developmental rates of 100 oocytes in each group.

### Statistical analysis:

All analyses were done at least in triplicate and data were reported as mean± SD. Statistical analyses were carried out using SPSS software version 23.0 (SPSS Inc., Chicago, IL, USA). One-way ANOVA and Kruskal-Wallis tests were respectively used for parametric and nonparametric variables. Tukey's H

SD was used as the post hoc test after one-way ANOVA. The p-value was set at ≤0.05 to determine statistically significant differences.

## Results

### The effect on ROS and GSH levels:

**Figure 1:**
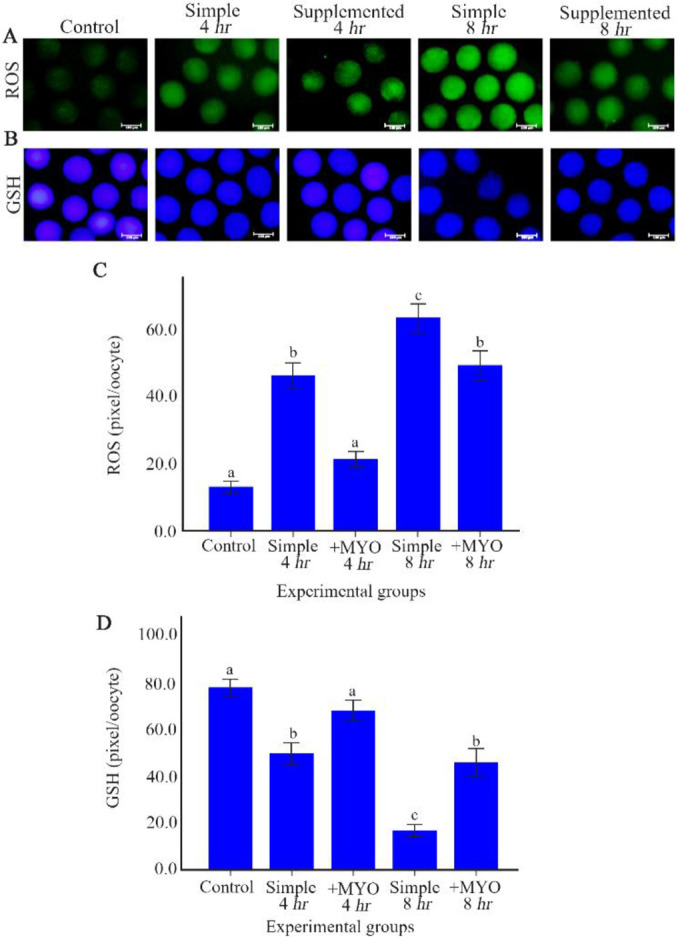
The effect of preincubation time and myo-inositol supplementation on the ROS and GSH levels in mouse MII oocytes. MII oocytes preincubated in each simple and supplemented medium for 0, 4 and 8 *hr* were dyed with (A) H2DCFDA and (B) Cell Tracker Blue to detect ROS and GSH levels, respectively. Scale bar indicates 100 *μm*. (C) The fluorescent intensity of MII oocytes stained by H2DCFDA and (D) Cell Tracker Blue. Fluorescent intensities of each stained oocyte were quantified with Image J software (ANOVA, p<0.001; Tukey’s post hoc, p<0.001)

The mean fluorescent intensity of ROS in preincubation groups except supplemented group with 4 *hr* of preincubation time was higher than control group (p< 0.001). Similarly, GSH mean intensity was higher in the control and supplemented group with 4 *hr* preincubation compared to the other groups (p< 0.001). Among the preincubation groups, oocytes which were exposed to 8 *hr* preincubation in the simple medium had the highest ROS and the lowest GSH intensity. Supplementation of MYO in the culture medium could reduce the intensity of ROS and lead to the augmentation of GSH intensity compared to the simple medium with both 4 and 8 *hr* of preincubation times. Interestingly, the intensity of ROS and GSH in the supplemented groups with 4 and 8 *hr* of preincubation was respectively similar to the control and simple group with 4 *hr* of preincubation (Table 1, Figure 1).

**Table 1:** Descriptive statistics of the oxidative stress, mitochondrial alteration, fertilization and developmental developmental rates in the mouse MII oocytes preincubated at different times and in different media

**Variables**	**No of oocytes**	**Control**	**4 *hr* preincubaton**		**8 *hr* preincubation**		**ANOVA/kruskal wallis p-value**

			**Simple medium**	**Supplemented medium**	**Simple medium**	**Supplemented medium**	
**ROS level (pixel/oocyte)**	40	13.01±1.78	47.17±3.79	20.26±2.25	63.19±4.29	49.24±4.29	˂0.001
**GSH level (pixel/oocyte)**	40	77.29±3.57	49.39±4.76	67.67±4.28	16.73±2.65	43.41±5.87	<0.001
**Amount of mitochondria (pixel/oocyte)**	40	80.16±1.64	60.34±4.63	79.90±4.99	44.90±6.04	57.00±2.81	<0.001
**MMP (red/green ratio/oocyte)**	40	0.50±0.07	0.46±0.01	0.47±0.02	0.33±0.02	0.35±0.03	<0.001
**ATP content (*ng/ml* )**	45	106.25±10.33	109.58±14.01	141.41±6.21	106.08±15.10	159.41±9.64	<0.001
**Fertilization rate**	100	84.64±4.02	78.90±1.11	82.95±3.55	66.92±2.66	77.23±2.02	<0.001
**Cleavage rate**		32.05±0.64	25.44±1.64	31.35±1.12	16.92±1.16	18.89±0.68	<0.001

ROS: Reactive oxygen species, GSH: Glutathione, ATP: Adenosine triphosphate. Results are presented as mean±SD

### The effect on the amount of mitochondria:

**Figure 2:**
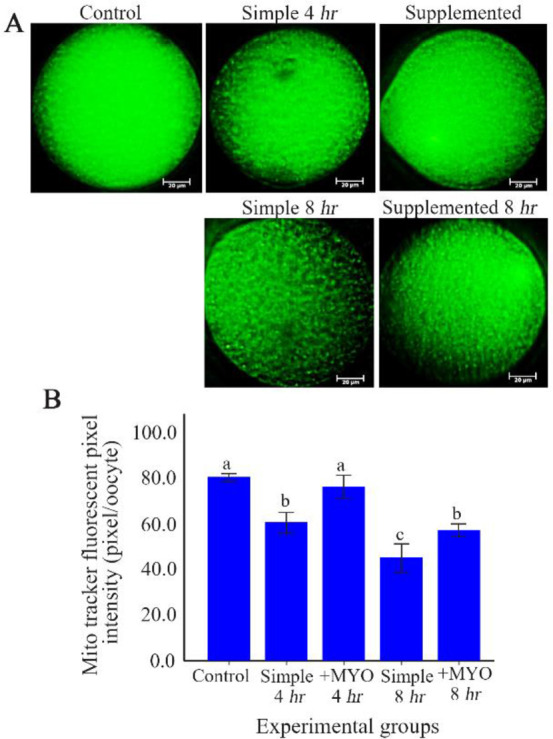
The effect of preincubation time and myo-inositol supplementation on the amount of mitochondria in mouse MII oocytes. A) MII oocytes preincubated in each simple and supplemented medium for 0, 4 and 8 *hr* were dyed with Mito Tracker Green to determine amount of mitochondria. Scale bar indicates 20 *μm*. B) The fluorescent intensity of MII oocytes stained by Mito Tracker Green. Fluorescent intensities of each stained oocyte were quantified with Image J software (ANOVA, p<0.001; Tukey’s post hoc, p<0.001)

The amount of mitochondria in the oocytes of control and supplemented group with 4 *hr* of preincubation was different from other preincubation groups, so that they showed higher amount of mitochondria compared to the oocytes of the other groups (p<0.001). In accordance with the ROS and GSH changes, the mean Mito Tracker Green fluorescence intensity of both simple groups with 4 and 8 *hr* of preincubation time were lower than equivalent supplemented groups. The fluorescence intensity in the supplemented group with 4 and also 8 *hr* of preincubation was respectively similar to the control and simple group with 4 *hr* of preincubation (Table 1, Figure 2).

### The effect on MMP:

**Figure 3:**
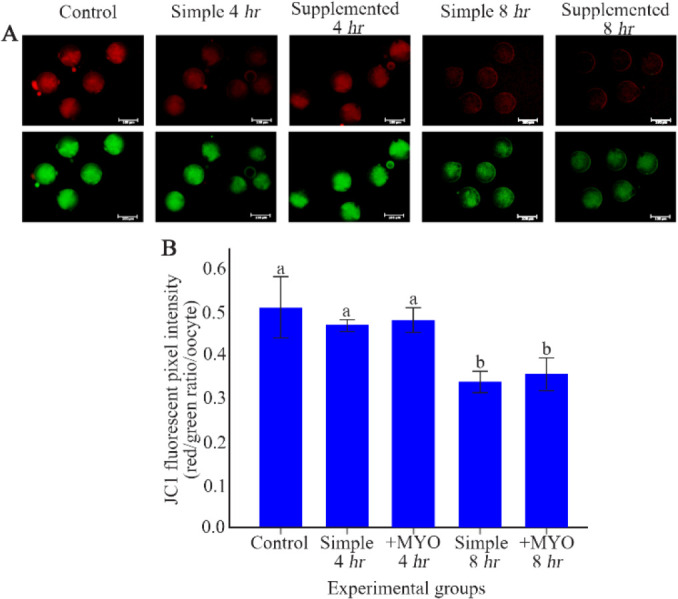
The effect of preincubation time and myo-inositol supplementation on the mitochondrial membrane potential in mouse MII oocytes. A) MII oocytes preincubated in each simple and supplemented medium for 0, 4 and 8 *hr* were dyed with JC-1 to detect mitochondrial membrane potential. Scale bar indicates 100 *μm*. B) The fluorescent intensity of MII oocytes stained by JC-1. Red/green fluorescent intensities of each stained oocyte were quantified with Image J software (ANOVA, p<0.001; Tukey’s post hoc, p<0.001)

In order to determine the mitochondrial membrane potential, the ratio of red/green fluorescence was measured. Unlike previous results, oocytes of control and both simple and supplemented groups with 4 *hr* of preincubation, showed similar levels in terms of mitochondrial membrane potential. MMP was decreased in simple/supplemented groups with 8 *hr* of preincubation compared to the control and groups with 4 *hr* of preincubation (p<0.001). The supplemented group with 8 *hr* of preincubation displayed equal MMP ratios when compared to the simple group with 8 *hr* of preincubation (Table 1, Figure 3).

### The effect on ATP content:

**Figure 4:**
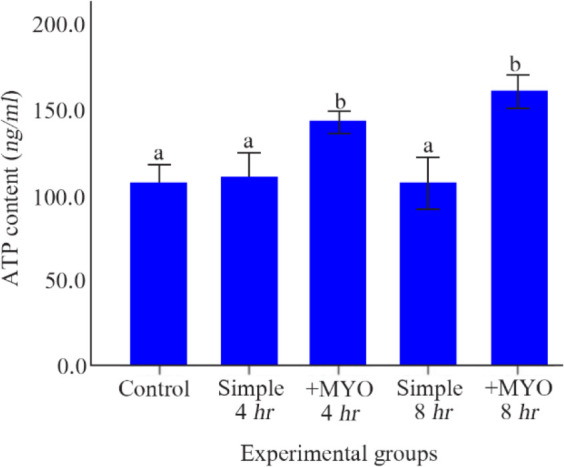
The effect of preincubation time and myo-inositol supplementation on the adenosine triphosphate (ATP) content. ATP content of mouse MII oocytes preincubated in each simple and supplemented medium for 0, 4 and 8 *hr* were analyzed by ELISA method. ATP content was calculated by using the formula derived from the linear regression of the standard curve (ANOVA, p<0.001; Tukey’s post hoc, p

However, ATP content in control and simple groups with 4 and 8 *hr* of preincubation was similar; the supplemented groups with 4 and 8 *hr* of preincubation showed an increase in ATP content in comparison to the control and simple preincubation groups (p< 0.001). Oocytes of supplemented groups with 4 and 8 *hr* of preincubation displayed equal contents of ATP (p˃0.05) (Table 1, Figure 4).

### The effect on the oocyte fertilization and developmental potential:

**Figure 5:**
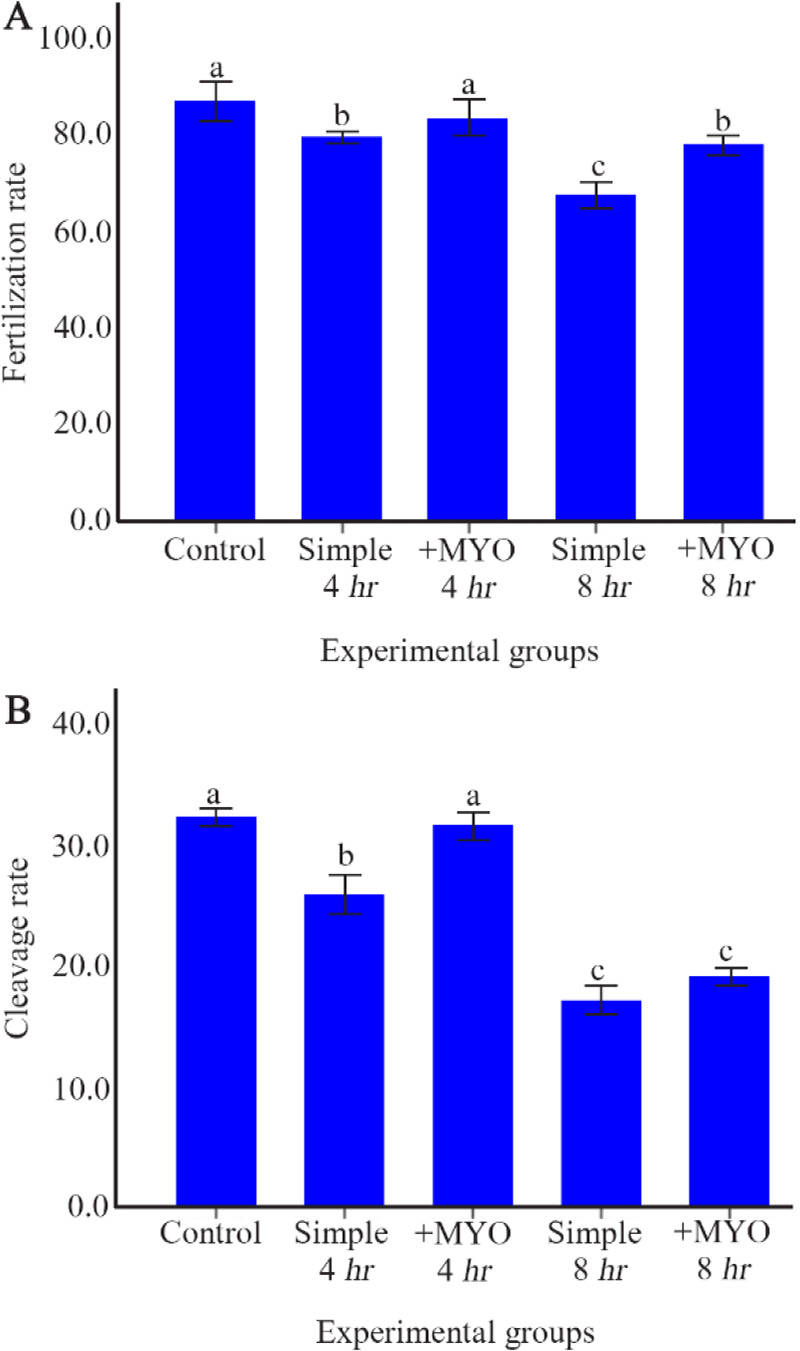
The effect of preincubation time and myo-inositol supplementation on Fertilization and developmental rates in mouse MII oocytes. A) Fertilization and B) developmental rates of mouse MII oocytes preincubated in simple and supplemented medium for 0, 4 and 8 *hr* were assessed respectively 4–5 *hr* and 3 days after co-incubation with sperm (ANOVA, p<0.001; Tukey’s post hoc, p<0.001)

It was found that Fertilization and developmental rates in the control and supplemented 4 *hr* preincubation groups were higher than other preincubation groups (p<0.001). In accordance with the pattern of changes in the ROS, GSH and amount of mitochondria, both supplemented groups with 4 and 8 *hr* of preincubation had higher Fertilization rate compared to the equivalent simple groups. The Fertilization rate in the supplemented group with 4 and also 8 *hr* of preincubation were respectively similar to the control and simple group with 4 *hr* of preincubation. Whereas myo-inositol supplementation could particularly increase developmental rates in the 4 *hr* preincubation group, it did not significantly improve developmental rate in the 8 *hr* preincubation group (Table 1, Figure 5).

## Discussion

It has been proven that postovulatory oocyte aging reduces oocyte quality and its developmental potential (14, 16). The present study investigated oocyte cellular and molecular changes and its fertilization potential following 4 and 8 *hr* of preincubation. The results of this study revealed that preincubation for 4 and 8 *hr* could decrease oocyte quality related to the elevation in the intracellular ROS levels, the decrease in GSH levels, altered amount of mitochondria and impaired mitochondrial membrane potential. MYO supplementation relieves these alterations and consequently improves fertilization potential.

This study showed that the control group had significantly lower ROS levels in comparison to the other preincubated groups, except supplemented group with 4 *hr* of preincubation time. Takahashi et al. in 2009 reported that the levels of ROS following 6 *hr* of incubation are higher than the one in mouse fresh oocytes (15). ROS are important mediators of intracellular signaling involving in numerous cell functions, including Ca^2+^ homeostasis and InsP3 receptor functions. Therefore, ROS production in aged oocytes might influence Ca^2+^ homeostasis directly and impair mitochondrial function (44). MYO supplement as an antioxidant which exists naturally in the follicular fluid (34) could alleviate ROS levels in the oocytes preincubated for 4 and 8 *hr* and preserve 4 *hr* in favor of oocytes. On the other hand, as expected, GSH levels as a ROS scavenger, were changed in a pattern that was completely opposite although was consistent with the ROS changes. Preincubation for 4 *hr* in the simple culture medium and for 8 *hr* in both simple/supplemented culture medium decreased the GSH levels significantly compared to the control group. Consistently, Boerjan and De Boer reported that the levels of ‘GSH decrease in 12 *hr* aged mouse oocytes (45). Decreasing GSH levels along with the elevated ROS levels make the aged oocytes prone to oxidative stress (21). Use of MYO antioxidant as a medium supplement could promote GSH levels in both 4 and 8 *hr* preincubation groups.

It has been indicated that, oocyte aging diminishes mitochondrial integrity which compromises the functionality of the mitochondria in the oocytes. The loose organelle arrangement may be caused by the high levels of oxidative stress induced by apoptosis (46). The present study suggested that fluorescence intensity due to the mitochondrial staining with Mito Tracker fluorochrome was significantly low in the groups with 4 and 8 *hr* of preincubation when compared to the control group. Nonetheless, addition of 20 *mmol/L* of MYO could improve the fluorescence intensity in both 4 and 8 *hr* preincubation groups and preserve 4 *hr* for preincubated oocytes.

Furthermore, in aged oocytes, oxidative stress induces shrinkage and dysfunction of mitochondria that reduces mitochondrial membrane potential. Consistently, results of the present study indicated that MMP after preincubation for 8 *hr* was lower than control group, although there was no difference between the MMP levels of oocytes preincubated for 4 *hr* and control group. Oocytes seem to enjoy a mitochondrial membrane potential stability to prevent excessive MMP loss and the consequent deleterious effects (47). Considering myo-inositol’s antioxidant effect (32) and its role in the mitochondrial membrane stability (25), it could increase MMP in both groups with 4 and 8 *hr* of preincubation compared to the equivalent simple groups; however, the differences were not statistically significant. Impaired mitochondrial membrane potential which is resulted from oxidative stress, is closely related to a decrease in ATP generation and oocyte quality (17, 22, 48, 49).

As predicted from the pattern of mitochondrial membrane potential change (22), ATP content was not altered after 4 and 8 *hr* of preincubation. It has been demonstrated that a decrease in the mitochondrial membrane potential and low ATP production are the final results of mitochondrial dysfunction (50) and 4 or 8 *hr* of preincubation might not be enough for occurrence of these changes. On the other hand, the mammalian mature oocyte can be aided by alternative salvage pathways of ATP production (51). The addition of MYO supplement in the culture medium increased the ATP content following 4 and 8 *hr* of preincubation compared to the control and simple groups with 4 and 8 *hr* of preincubation. MYO has also been suggested to play an important role through the activation of phospholipase C, resulting in the production of inositol triphosphate (IP3) (InsP3) and calcium channels opening. This mechanism induces Ca^2+^ release from internal stores and consequently, increases intracellular concentrations in the cell. The activation of these intracellular mechanisms induces an increase in the cytosolic calcium and consequently an enhancement in themitochondrial Ca^2+^ stimulating the oxidative meta-bolism and the ATP production (52, 53).

Interestingly, the pattern of fertilization and developmental potential changes between the groups was similar to the changes in the ROS, GSH levels and amount of mitochondria. According to the studies, oxidative stress and mitochondrial heterogeneity without mitochondrial malfunction are sufficient to reduce the oocyte fertilization and developmental potential (54, 55). In the same way, the alleviation of oxidative stress with myo-inositol supplement can improve the fertilization potential. Noticeably, the medium supplementation can improve developmental potential until 4 *hr* of preincubation and is not effective in the embryos produced from 8 *hr* preincubated oocytes. Myo-inositol was unable to improve the two variables of mitochondrial membrane potential and developmental rate in the 8 *hr* preincubated oocytes. This may be a justification for the probable impact of the mitochondrial potential alteration, as a late time- dependent change, on the development potential of the oocytes (56). It is important to note that prolonged oocyte culture is associated with other molecular and cellular changes, especially spindle alteration. Spindle changes are associated with the chromosome segregation and its assessment can also be helpful in determining the optimal oocyte preincubation time (14, 17, 57).

## Conclusion

This study evaluated cellular and molecular changes in the oocyte following preincubation and showed that even a 4 and/or 8 *hr* preincubation time can lead to alteration in the ROS, GSH levels, amount of mitochondria, mitochondrial membrane potential, fertilization and developmental rates. It was demonstrated that oocyte culture medium supplemented with 20 *mmol/L* of myo-inositol effects the ROS and GSH levels, improves amount of mitochondria and increases fertilization and developmental rates following 8 *hr* of or less preincubation time. Interestingly, MYO supplementation caused equivalent results in control and supplemented 4 *hr* preincubation groups and also in simple group with 4 *hr* of preincubation and supplemented 8 *hr* preincubation group and preserved 4 *hr* in favor of oocytes.
